# Physical Characteristics and Performance Tests in Male Water Polo: A Multiple Regression Analysis on Youth

**DOI:** 10.3390/ijerph19148241

**Published:** 2022-07-06

**Authors:** Giovanni Melchiorri, Tamara Triossi, Daniele Bianchi, Virginia Tancredi, Valerio Viero

**Affiliations:** 1 Department of Systems Medicine, School of Sport and Exercise Sciences, Faculty of Medicine and Surgery, University of Rome Tor Vergata, Via Montpellier 1, 00133 Rome, Italy; gmelchiorri@libero.it (G.M.); tancredi@uniroma2.it (V.T.); 2 Don Gnocchi Foundation IRCCS, Piazzale Rodolfo Morandi 6, 20121 Milan, Italy; 3 Italian Swimming Federation, 00135 Rome, Italy; tamaratriossi@hotmail.com (T.T.); danie-94@libero.it (D.B.); 4 School of Sport and Exercise Sciences, Faculty of Medicine and Surgery, University of Rome Tor Vergata, Via Montpellier 1, 00133 Rome, Italy; 5 Centre of Space Bio-Medicine, University of Rome Tor Vergata, Via Montpellier 1, 00133 Rome, Italy

**Keywords:** anthropometric characteristics, physical performance, match analysis

## Abstract

Background: In water polo, more physical and performance variables are related to a performance in a match. The aim of our work was therefore: (a) to evaluate the relationships between anthropometric characteristics and performance tests and performance in a match in young male water polo players; (b) to propose new guidelines for match analysis. Methods: Multiple regression analysis was used to study the results in anthropometric evaluations (height, body mass, chest circumference, arm span, non-dominant arm length) and performance tests (push-up, chin-up, shuttle swim test, sprint swim 10 m, eggbeater kick, 100 m swimming) and two coaches’ evaluations of two friendly matches using new guidelines. A total of 130 subjects (age: 15.6 ± 0.9 years) were involved in the study. Results: In this study, we proposed a new performance model based on multiple regression analysis (r = 0.85, r^2^ = 0.73, adjusted r^2^ = 0.57) and described by the following equation: Coach’s Evaluation = 151.6 + (−0.016 × height) + (0.6 × body mass) + (−0.82 × chest) + (−0.59 × arm span) + (0.75 × non dominant arm length) + (−0.037 × push up) + (0.17 × chin up) + (5.87 × shuttle swim test) + (−2.2 × 10 m sprint swim) + (0.05 × eggbeater kick) + (−0.35 × 100 m swimming). Inter-observer values were: CV: −3.9%, ICC: 0.82, ES: 0.1. Intra-observer: CV: −4.1%, ICC: 0.96, ES: 0.06. Conclusions: The relationships between anthropometric and performance variables and the match analysis have been statistically described. The equation found can be used to predict the overall performance of a player and permits evaluations of how much the improvement in one of the qualities can affect the players’ overall performance. Moreover, the new method for match analysis we have proposed showed a good reliability and can be used for new studies on water polo.

## 1. Introduction

In high-demanding team sports such as basketball [1], rugby [2], and handball [3], anthropometric and fitness performance characteristics have been associated with a greater possibility for young athletes to grow into elite adult players. Among team sports, water polo is definitely a high-demanding one as it is characterized by organized physical contact among players in which anthropometric characteristics play a most important role, as pointed out by various authors [4,5,6,7]. Analyzing swimming capacity in water polo [8,9], other authors have observed that even a better swimming performance is related to the general performance level of a young water polo player.

According to some researchers [10], neuromuscular efficiency can be related to swimming performance; due to this reason as well as the presence of physical contact among players, it would seem reasonable to also use dry land tests to evaluate the characteristics of young water polo players. In water polo, as in other high-demanding sports, more physical and performance variables are therefore related to a performance in a match. Sport-specific performance, or the match, can therefore be considered as an output variable influenced by a complex combination of several other factors.

Actually, we were able to find only two water polo studies on the association between anthropometric and conditioning capacities and efficacy in different situations in young water polo players. The first [8] studied the associations with offensive and defensive action abilities, and the other one [11] with specific skills. Neither of the two studies treated the overall analysis of the match and the coach’s evaluation of young players’ capacities. The aim of our work was therefore to use multiple regression analysis to study the association between the overall assessment of the single athlete made by the coach throughout the match (predict) and some variables (predictors). An ancillary aim was to introduce a system for match analysis following guidelines that can be used by coaches to provide an objective overall evaluation of the player during the match.

## 2. Materials and Methods

### 2.1. Subjects

A total of 136 subjects were recruited but only 130 respected the inclusion criteria and participated in the study. The inclusion criteria were: (1) participate in a national youth water polo championship of the national federation; (2) be male and aged between 14 and 17; (3) having participated in a regional or national process of talent identification and selection with at least 60% attendance; (4) have not suffered from injuries in the last 6 months; (5) have not ever played in first and second national division championships. The subjects recruited (age: 15.6 ± 0.9 years; height: 171.2 cm ± 8.3; body mass: 61.7 ± 9.5 kg) were part of a project for talent identification and selection to the water polo National Team. The sample was then randomly divided into two sub-groups: “measured” and “estimated”. The “measured” group included 65 young water polo players (age: 15.7 ± 0.7 years; height: 171.7 ± 7.4 cm; body mass: 61.5 ± 8.8 kg). The “estimated” group included 65 subjects (age: 15.4 ± 1.1 years; height: 170.6 ± 9 cm; body mass: 62.0 ± 10.1 kg).

### 2.2. Experimental Procedure

**Figure 1:**
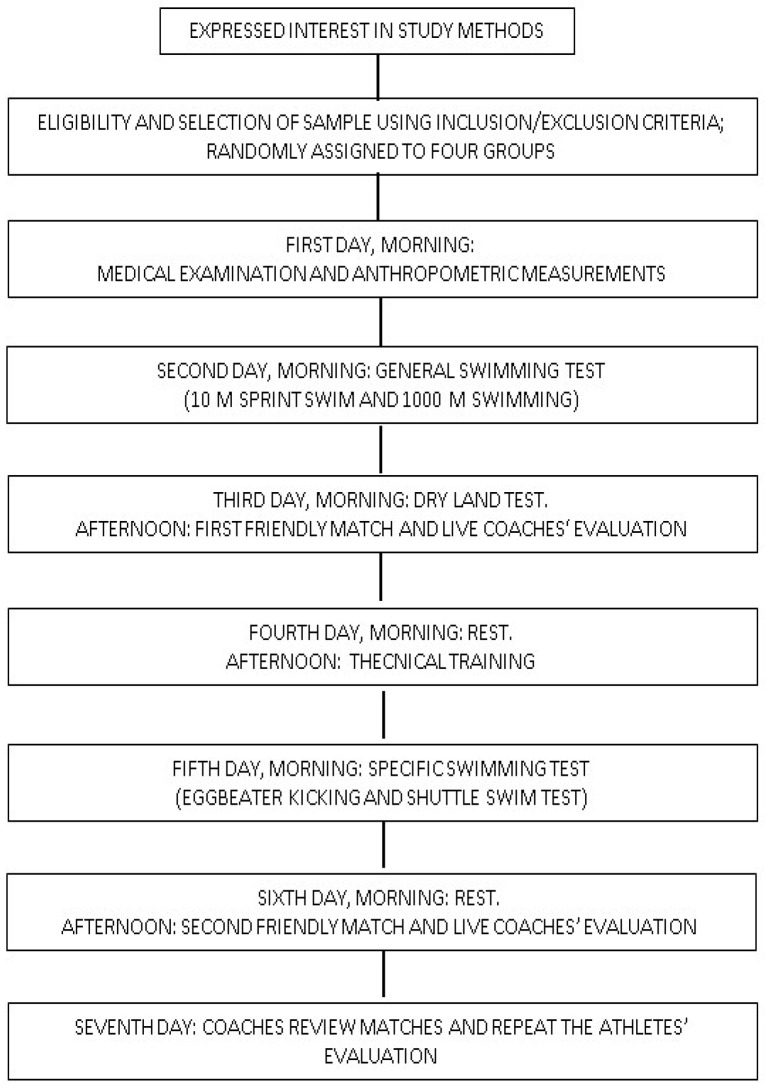
Consort diagram: sequence of study assessments.

In the middle of the season, when clubs’ training programs were well-established, players from across the nation were summoned in different groups of 26 players to a 6-day meeting for each group. On the first day, anthropometric measurements (body mass and height, chest circumference, arm span, non-dominant arm length) were collected, and all the players underwent medical examinations to exclude the presence of current pathologies or recent traumatic events. General swimming tests (10 m sprint swim and 100 m swimming) were performed on the second day, while dry land tests (push-ups and chin-ups) were performed in the morning, and the first friendly match was held on the afternoon of the third day. The fourth day included rest in the morning and technical training in the afternoon, while specific swimming tests (the eggbeater test and shuttle swim test) took place on the fifth day. The last day comprised a half day of rest in the morning and a second friendly match in the afternoon. The adopted training scheme was comparable to what the players would regularly do with their own clubs. In Figure 1, the sequence of the study assessments is shown by a consort diagram.

At the end of the matches, the two coaches provided a first evaluation for each individual player based on live observation (T0). After seven days, they reviewed the match records and provided a rating for each single player (T1). For the evaluation of the repeatability of the measurements performed, both intra-observer variability (comparisons between the measurements made at T0 and those made at T1 by each technician) and inter-observer variability were considered. As regards the intra-observer repeatability study, we analyzed variability between the observations the technicians had made apart from each other. The inter-observer study instead required a comparison between the data recorded by S1 and S2.

### 2.3. Anthropometric and Body Mass Measures

#### 2.3.1. Body Mass and Height

Body mass was measured using a 100 g precision electronic scale (Sea, Berlin, Germany) under standard conditions (fasting in the morning, subjects unclothed and without shoes). Height was measured with a 1 mm precision wall ruler (Sea, Berlin, Germany).

#### 2.3.2. Circumferences and Lengths

Chest circumference, arm span (total upper-limb width), and non-dominant arm length measurements were carried out according to conventional criteria and measurement procedures [12].

### 2.4. Performance Test

#### 2.4.1. 10 m Sprint Swim

The objective of the test, performed over a 10 m distance, was to assess short-distance speed capacities. The starting position was the same as in the sprint for ball possession at the start of the game, and a whistle signal was given for the subjects to initiate the test. No specific head position was required during swimming. A total of three trials were performed, and the best was considered for further analysis [8]. 

#### 2.4.2. 100 m Swimming

The objective of the test was to assess short-distance swimming capacities. The test was carried out in a 25 m pool: the players were permitted to push off the wall, but in order for the test to be homogeneous, flip-turns were not allowed [8].

#### 2.4.3. Eggbeater Test (Eggbeater)

The test measures lower-limb efficiency through the action of the eggbeater kicking movement in a vertical position and the use of an overload set at 10 kg for all subjects. Endurance in the vertical position was measured until exhaustion [13].

#### 2.4.4. Shuttle Swim Test

The result of the shuttle swim test is a measure of water polo players’ ability to move effectively in water. The test result we considered the most was the average speed in ms^−1^ over a 240 m swimming trial, made up of 10-to-40-m trials with changes in direction and posture [9].

#### 2.4.5. Chin-Up

Chin-ups were performed with a supinated, shoulder-width grip, starting with arms bent and the chin above the bar [4]. 

#### 2.4.6. Push-Up

Push-ups were performed starting from a prone position, with extended arms and hands resting on the ground at a wider-than-shoulders distance [4]. 

In both the chin-up and push-up tests, we counted complete repetitions carried out until exhaustion. 

### 2.5. Coach’s Evaluation

Each coach (S1 and S2) observed the live games on their own and watched the video recordings of the same games after seven days. As suggested by Maynard for other team sports, we adopted a decision-making-ability evaluation scheme adapted to water polo to guide the coaches’ assessments [14,15] since decision-making performance is considered of the utmost importance in water polo [16] and in team sports in general.

To prepare themselves for the evaluation and get an updated and accurate idea of the professional standards, the technicians had watched all the water polo matches of the last Olympic Games. The evaluation of each young player was made using a 0–10 scale (1: recreational playing standard; 10: professional playing standard). The recorded score can be multiplied by 10 and expressed as a percentage to facilitate its use by coaches. In professional-level players, we expect a rating of 9–10 (90–100%), and in recreational-level players, 1–2 (10–20%). Afterwards, they compared the decision-making-ability levels of the observed players, considering their resemblance to the standards of professional players. The evaluation was conducted by adopting a “gain of possible gain” logic and expressed as a percentage [14]. The guidelines for the evaluation based on what Maynard proposed for football and here adapted to water polo are shown in Table 1 [14,15].

The operators had overlapping skills and experiences (they were both water polo coaches, graduates in motor science, and had received the same training for the measurements required by the study).

### 2.6. Statistics

The null hypothesis of our study was that the overall evaluation of single players was not influenced by anthropometric and performance variables, whereas according to the alternative hypothesis, the assessment was influenced by several variables related to body structure and conditioning capacities.

Data were recorded using Excel 16.3 (Microsoft, Redmond, WA, USA) and analyzed with the SPSS 21 software (IBM Inc., Armonk, NY, USA), and are reported as means and standard deviations. The normality of the data was tested by means of normality plots and Kolmogorov Smirnov tests. Levene’s test was used to investigate the homogeneity of variance.

The following tests were adopted for the assessment of the reliability of the Coach’s Evaluation. The coefficient of variation (CV) was calculated using the following formula: (SD/M) × 100, with SD being the standard deviation and M the mean difference. The measurement error (ME) was calculated as SD/√2 and the error range as ME × critical value. The critical value was fixed at 1.96 [17]. SEM (standard error of measurement) was calculated by the formula: SEM = SD/√*n*, with *n* being the size of the sample under examination. The SEM error range was calculated as SEM × critical value. The critical value was fixed at 1.96. SEM was employed to estimate the reliability and minimal detectable difference [18]. Cohen’s d effect size was used to study the effect size according to the formula M1-M2/SD pooled, with M1 being the mean value of the first measurement, M2 the average value of the second, and SD the standard deviation. An intraclass correlation coefficient (ICC) was used to evaluate the correlation between continuous variables for intra- and inter-observer differences, as well as between the measured and estimated Coaches’ Evaluations [18]. The reliability analysis was completed by using a Bland and Altman plot.

The following tests were used for multiple regression analysis. An ANOVA analysis was conducted to test the effectiveness of the proposed model. The Durbin–Watson test was used to verify the serial correlation between errors, and therefore the assumption of independence between the variables and the accuracy of the model.

## 3. Results

### 3.1. Players

Table 2 shows the data recorded on our sample (130 subjects), reporting anthropometric data, performance tests in water, and those performed on dry-land. Overall, the players had an assessment of their decision-making ability, according to the Coach’s Evaluation, of 36.1 ± 10.5 (95% CI: 34.3–38.1).

### 3.2. Coach’s Evaluation Reliability

**Figure 2:**
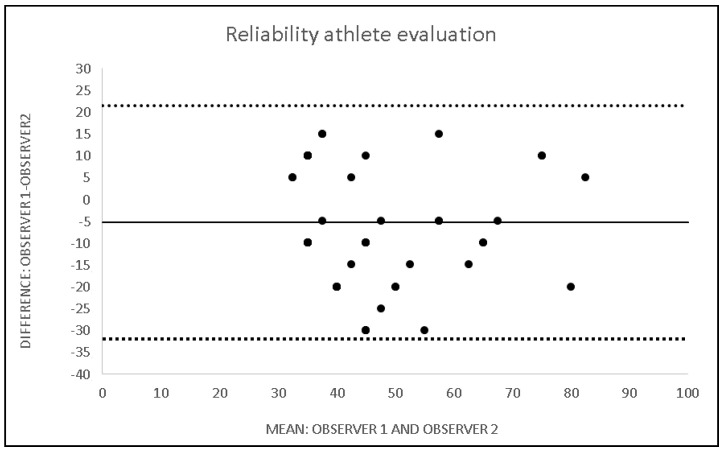
Inter-observer differences.

An analysis of the inter-observer differences revealed the following values: coefficient of variation: −3.9% (ranged 0.2–1.9), intraclass correlation coefficient: 0.82 (lower bound 0.75; upper bound: 0.87), effect size: 0.1, and measurement error: 6.7 (error range 13.2). The standard error of measurement has a value of 0.84 (error range 1.6). In 75 out of the 130 total cases (57%), the evaluations of the 2 observers had the same value. To further investigate the reliability of the method, a graphical statistical analysis was performed according to Bland and Altman on the remaining 55 subjects with differences between S1 and S2 (Figure 2).

Intra-observer analysis showed the following values: coefficient of variation: −4.1% (range 0.2–1.4), intraclass correlation coefficient: 0.96 (lower bound: 0.95; upper bound: 0.99), effect size: 0.06, and measurement error: 3.4 (error range 6.7); the standard error of measurement = 0.45 (range: 0.86).

### 3.3. Model

The adopted model considers as predictive variables: height (the player’s height, cm), body mass (the player’s body mass, kg), circumference of chest (circumference of the player’s chest, cm), arm span (distance between hands, cm), non-dominant arm length (non-dominant arm length of the player, cm), push-up (max number of push-ups performed), chin-up (max number of chin-ups), shuttle swimming test (speed in the shuttle swimming test, ms^−1^), sprint over 10 m (time recorded during a 10 m sprint, seconds), eggbeater test (time to exhaustion in eggbeater kicking in vertical position, seconds) and swimming test over 100 m (time recorded during a 100 m swim at maximal intensity, seconds). Table 2 shows the values relating to the analysis of the model obtained with multiple regression analysis. The equation of our model is reported below. The model summary was: r value = 0.85; r^2^ value = 0.73; adjusted r^2^ = 0.57; Durbin-Watson = 2.3; F ratio = 2.5; significativity = 0.01.

Coach’s Evaluation = 151.6 + (−0.016 × height) + (0.6 × body mass) + (−0.82 × chest) + (−0.59 × arm span) + (0.75 × non-dominant arm length) + (−0.037 × push up) + (0.17 × chin up) + (5.87 × shuttle swim test) + (−2.2 × 10 m speed swim) + (0.05 × eggbeater) + (−0.35 × 100 m swimming). In Table 3, the multiple regression results of the independent variables are summarized.

**Figure 3:**
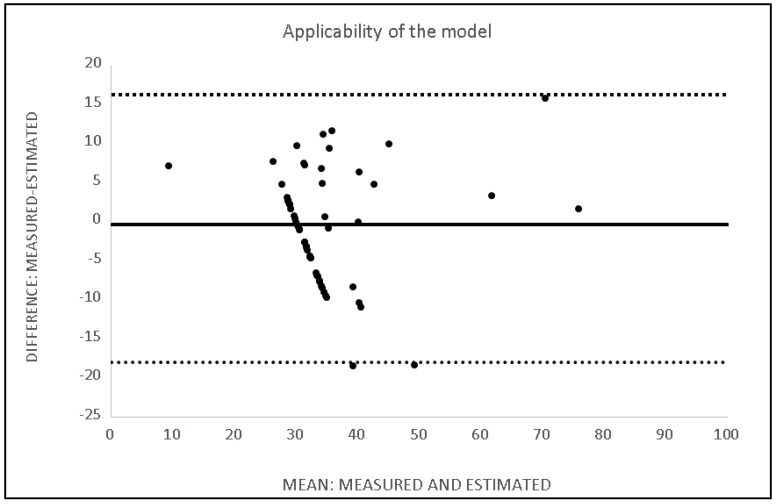
Bland and Altman plot describes the reliability of the model. The measured value is the value directly provided by using the Coach’s Evaluation, and the estimated value is the value obtained from the application of the model.

The “measured” sub-group reported an average rating of the technicians of 34.4 ± 10.3 (95% CI: 31.7–37.0), and the “estimated” group had average values of 35.1 ± 9.8 (95% CI: 32.6–37.7) The data measured directly from the “estimated” subgroup were compared with those estimated for the same group starting from the predictor variables. The coefficient of variation is −8.8%; total error: 6.8 arbitrary unit (AU) (error range 10.8 AU); effect size: 0.1; intraclass correlation coefficient 0.86 (lower bound 0.77 and upper bound 0.92); standard error of measurement: 0.89 (error range 1.74). Figure 3 shows the Bland and Altman plot of the reliability of the model.

## 4. Discussion

### 4.1. Players

The average value for the Coach’s Evaluations recorded on our overall sample is 36.1 ± 10.5, which shows, as expected, that within the considered age group, the young water polo players’ level of preparation is still lacking in this respect. Youth’s motor skills and trainability is very much influenced by their maturation level [19], and in an open skill sport as complex as water polo, it is conceivable that our young champions had not yet reached the highest levels of motor skills and abilities on which decision-making abilities are based. From this perspective, a limitation of our study is the fact that the subjects involved all belonged to the same age group; longitudinal studies on different age groups are therefore required for further analysis. Some of the anthropometric and performance variables considered here were also used by other authors to conduct studies on young water polo players [8,20]. 

### 4.2. Coach’s Evaluation

Water polo is an open skill sport, and lacking any standardized method to analyze decision-making ability, we created a new one based on what had been done in other sports and studied its validity in terms of inter-observer and intra-observer reliability.

Inter-observer differences are trivial as demonstrated by the values for the coefficient of variation, intraclass correlation coefficient, effect size, and measurement error, and also suggests a good applicability of the method of analysis in longitudinal studies on young players. Good statistical results were also produced for inter-observer reliability. The same score was obtained by the 2 observers in 57% of the subjects evaluated. In the remaining cases, we studied repeatability using a Bland and Altman plot (Figure 2). The plot shows only a small amount of random error that is scattered around the line of no difference and that the differences are within the upper and lower bound. The evaluation system therefore seems to demonstrate good inter- and intra-operator reliability on the population examined.

The standard error of measurement provides information on the error measurement and can also be useful for calculating the minimal detectable difference [18]. The SEM’s value is almost double in the evaluation of the inter-observer differences (0.84) compared to that calculated for the intra-observer differences (0.45), thus indicating that for repeated studies over time, an evaluation by a single operator is to be preferred.

Regarding the sample examined in our investigation, the coefficient of variation, effect size, intraclass correlation coefficient, and standard error of measurement show a good applicability of the coaches’ evaluation of decision-making abilities. In the investigated age group, water polo confirms to be a sport in which anthropometric characteristics and physical performance play an important role in determining a player’s value in the eyes of the technician. It is confirmed that a player’s capacities in the game of water polo can be improved by good physical training and that some physical characteristics in young water polo players are associated with better performance and can be considered as a prerequisite, or as a tool for effective decision making. The performance variables can be best trained because they are more modifiable (swimming, strength), while the anthropometric should be sought in the process of identification and selection of talent (height, arm span, limb length).

### 4.3. The Model

Water polo is a very physically demanding, organized contact sport, and several authors indicate physical abilities and body size as important requirements to achieve a good level of quality in technical–tactical actions, both for young and adult players [4,5,6,7,8,9,20,21]. As evidenced by these studies, the player’s performance in water polo and team sports in general [1,2] has a multifactorial origin, and multiple regression analysis can be used to analyze the relationships between some predicting variables (independent predicting variables) and a predicted one. In other team sports [1], multiple regression analysis has been applied using the performance during the match as an output variable (predict) and anthropometric variables and performance evaluation as the predicting variable. As regards water polo, only two papers applied multiple regression analysis, with one using as the predictor the effectiveness measured during some actions of the match (attack and defense) instead of an overall analysis of the same [8]. The other one examined the associations of players’ specific skills with their anthropometric and general motor characteristics in a sample younger than ours (12–14 years) [11].

In relation to the multifactorial nature of water polo, a good general swimming efficiency is necessary to move continuously and quickly from one part of the field to the other and in “stop and go” actions [5,8,9,22]. Good muscle strength of the upper limbs is advantageous in the contact phases with the opponent as well as in shooting [23]. The lower-limb efficiency with the eggbeater kick movement is important to maintain the correct vertical position while throwing and passing the ball [12], just as the peculiar physical characteristics facilitate swimming and technical actions [6,20], improving the trainability of water polo players. This is why these variables were chosen.

As far as a general concept of trainability [24] is concerned, given the demonstrated multifactoriality of water polo performances, we chose the predictor variables to be used in the multiple regression analysis considering both anthropometric variables and in-water and dry-land performance variables. The model we developed therefore includes 11 variables describing physical and performance characteristics chosen according to a theoretical criterium [24] and the expert knowledge of water polo coaches and physical trainers. In the multiple regression analysis, the model takes the form of an equation, and the coefficient for each variable tells us about the relationship between the Coach Evaluation of the match performance and the predictor when other variables are held constant, so the equation can be used to predict the match performance and the way the player’s match performance as perceived by the technician can be modified when one of the variables changes (physical and performance characteristics, the predictors).

The model has a good efficacy (r: 0.85) and allows us to highlight that 73% of the match performance is explained by the anthropometric and performance variables proposed (r^2^ value = 0.73). This value is to be considered rather high since our model does not include psychological evaluations nor skill abilities. These would be expected to affect the match performance and the evaluation of young water polo players more than the residual 27%. The adjusted r^2^ (0.57) gives us some idea of how well the proposed model generalizes. The difference between r^2^ and the adjusted r^2^ provides information on the use of the model in the general population. In our case, the difference is not actually small, confirming that the model is very specific to the sample tested.

The assumption that independent errors are acceptable occurs when the Durbin Watson statistic is close to 2 and in any case between 1 and 3. In this case, we have a value of 2.3, which confirms the fitness of the model to our sample [25]. The F ratio (2.5) represents the ratio of improvements in predictions that result from fitting the model relative to the inaccuracy that still exists in the model itself, and helps us to understand how the proposed model is no doubt better than one using the mean of the predictors to measure the values for predict [25].

To confirm the usefulness of the model for predicting young water polo players’ performances, we compared within the “estimated” sub-group the result measured during the observation of the matches with that obtained using the model formula reported in Results. The Bland and Altman plot (Figure 3) confirms reliability between the measured and predict values obtained with the equation reported in Results. A small amount of random error is scattered around the line of no difference, and the differences fall within the upper and lower bound values.

## 5. Limitations of the Study

Water polo was born in Europe [26] and then developed around the world with different schools distinguished by anthropometric, physical, and technical differences, as well as different interpretations of the game. A limitation of the evaluation system could therefore be represented by the different ways of interpreting the game, and studies employing international samples are therefore desirable. As highlighted above, the model is very specific to the sample tested and cannot be used with different age, gender, or competitive-level samples [25]. Studies on these different groups would be therefore desirable as well. Other limitations of the study are the size of the sample and the number of the coaches involved, which could be larger.

## 6. Conclusions

The model suggests the possibility of improving the coach’s evaluation by also enhancing both anthropometric (more difficult to modify) and performance (more easily modifiable) variables. The investigated anthropometric variables of height, body mass, chest circumference, arm span, and non-dominant arm length are specific characteristics of young water polo players which, together with efficiency in physical performance tests (push up, chin up, eggbeater, shuttle swim test, 10 m sprint swim, 100 m swimming), help to explain the overall assessment of a young water polo player by a coach (see the Coach’s Evaluation equation). The equation can be also used to predict the overall performance of a water polo player. It permits the evaluation of how much the improvement in one of the qualities introduced in the model can affect the player’s total performance (allowing for time-saving and evidence-based training). The scale proposed for the decision-making-ability assessment can be used to perform a standardized analysis of the players and to monitor the improvements of the individual player, while also providing the player with feedback on the objectives to be pursued (improvement of training compliance).

The article proposes a new way to evaluate (using the scale) and predict (using the model) performances during a match. A coach can understand how much anthropometric variables and physical performance affect their overall evaluation of the player, thus separating them from the technical ones. This approach can help the coach to identify improvement points for each player (swim performance, dry land performance, technical abilities). With this method, the coach can also improve their talent identification methods.

## Figures and Tables

**Table 1:** Guidelines to the coaches’ evaluation of the players’ decision-making performance.

	Description
Rating	Professional Player Standard
9–10	Knows how, where, and when to improvise and move forward Knows how, where, and when to swim in order to create space for others teammates Can anticipate the opposition’s play when defending Gives verbal information to other players Knows how to delay two players Knows how, where, and when to feint or to shoot with efficacy
7–8	Knows how to behave in a static defense situation with an even number of players Knows how to behave in a defense situation with one less player Knows whether and how to support a teammate with ball possession in a positional attack situation Knows when and how to move wide Knows how to “check off” an opponent Knows where and when to feint or to shoot; the shoot is usually efficient
5–6	Knows when, where, and how to pass Knows when to pass the ball forward, square, and back Knows where and when to delay and tackle Knows when to swim or to hinder opponents Knows when to balance the defense Knows how to make a recovery swimming and when to mark opponents Knows where and when to dribble and when to shoot
3–4	Makes bad decisions about when, how, or where to pass Controls the ball in situations that demand first-time play Makes bad decisions about when or where to support Neither balances the defense, nor knows where to dribble Fails to perceive shooting opportunities
1–2	Makes bad decisions about when, how, and where to pass Makes bad decisions about when and where to support Neither provides width nor marks the opponents
	Recreational Player Standard

**Table 2:** Anthropometric and performance test for all players.

	Mean and SD	IC 95%
Height (cm)	171.2 ± 8.3	169.0–172.7
Body mass (kg)	61.7 ± 7.5	59.9–64.2
Chest circumference (cm)	88.0 ± 7.7	86.3–89.5
Arm span (cm)	175.4 ± 5.9	173.2–177.6
Non-dominant arm lenght (cm)	70.6 ± 7.1	69.3–71.9
Swimming 100 m (s)	70.2 ± 5.8	66–71.5
Swim speed 10 m (s)	6.4 ± 1.1	6.2–6.8
Shuttle swim test (ms^−1^)	1.4 ± 0.2	1.40–1.48
Chin up (number)	6.7 ± 4.4	5.5–7.6
Push up (number)	23.3 ± 11.2	20.4–25.8
Eggbeater test (s)	29.2 ± 6.0	19–32

**Table 3:** Multiple regression results of the independent variables. B: unstandardized regression coefficient.

		95% CI		
	B	Lower Bound	Upper Bound	*p*
Height	−0.16	−0.70	0.80	0.96
Body mass	0.60	0.05	1.10	0.03
Chest circumference	−0.80	−1.30	−0.30	0.00
Arm span	−0.60	−1.50	0.30	0.18
Non-dominant arm length	0.70	−0.30	1.80	0.15
Swimming 100 m	−0.30	−1.20	0.50	0.42
Swim speed 10 m	−2.20	−4.30	−0.10	0.03
Shuttle swim test	6.00	−10.00	21.00	0.45
Chin up	0.20	−0.50	0.80	0.61
Push up	−0.10	−0.30	0.20	0.77
Eggbeater test	0.05	−0.10	0.20	0.59

## Data Availability

All study data are included in the present manuscript.
